# Decreased hippocampal translocator protein (18 kDa) expression in alcohol dependence: a [^11^C]PBR28 PET study

**DOI:** 10.1038/tp.2016.264

**Published:** 2017-01-10

**Authors:** N J Kalk, Q Guo, D Owen, R Cherian, D Erritzoe, A Gilmour, A S Ribeiro, J McGonigle, A Waldman, P Matthews, J Cavanagh, I McInnes, K Dar, R Gunn, E A Rabiner, A R Lingford-Hughes

**Affiliations:** 1 National Addictions Centre, Institute of Psychiatry, Psychology and Neuroscience, Kings College London, London, UK; 2 Neuroimaging Department, Kings College London, London, UK; 3 Centre for Neuropsychopharmacology, Imperial College London, London, UK; 4 Division of Brain Sciences, Imperial College London, London, UK; 5 West London Mental Health NHS Trust, London, UK; 6 Centre for Infection, Inflammation and Immunity, University of Glasgow, Glasgow, UK; 7 Institute of Health and Well-being, University of Glasgow, Glasgow, UK; 8 Central and North West London NHS Trust, London, UK; 9 Imanova Limited, London, UK

## Abstract

Repeated withdrawal from alcohol is clinically associated with progressive cognitive impairment. Microglial activation occurring during pre-clinical models of alcohol withdrawal is associated with learning deficits. We investigated whether there was microglial activation in recently detoxified alcohol-dependent patients (ADP), using [^11^C]PBR28 positron emission tomography (PET), selective for the 18kDa translocator protein (TSPO) highly expressed in activated microglia and astrocytes. We investigated the relationship between microglial activation and cognitive performance. Twenty healthy control (HC) subjects (45±13; M:F 14:6) and nine ADP (45±6, M:F 9:0) were evaluated. Dynamic PET data were acquired for 90 min following an injection of 331±15 MBq [^11^C]PBR28. Regional volumes of distribution (*V*_T_) for regions of interest (ROIs) identified *a priori* were estimated using a two-tissue compartmental model with metabolite-corrected arterial plasma input function. ADP had an ~20% lower [^11^C]PBR28 *V*_T,_ in the hippocampus (F(1,24) 5.694; *P*=0.025), but no difference in *V*_T_ in other ROIs. Hippocampal [^11^C]PBR28 *V*_T_ was positively correlated with verbal memory performance in a combined group of HC and ADP (*r*=0.720, *P*<0.001), an effect seen in HC alone (*r*=0.738; *P*=0.001) but not in ADP. We did not find evidence for increased microglial activation in ADP, as seen pre-clinically. Instead, our findings suggest lower glial density or an altered activation state with lower TSPO expression. The correlation between verbal memory and [^11^C]PBR28 *V*_T_, raises the possibility that abnormalities of glial function may contribute to cognitive impairment in ADP.

## Introduction

Alcohol dependence can lead to cognitive impairment. Severe forms of alcohol-related brain damage are well recognized, but less often acknowledged is that even apparently functioning alcohol-dependent patients (ADPs) suffer from a range of cognitive deficits.^1, 2^ These include problems with memory and executive function that persists for months.^2^ Though there is improvement after one year of abstinence, these deficits affect the ability of patients to engage with after-care and to remain abstinent.^3, 4, 5, 6^

Multiple causal factors contribute to alcohol-related brain damage including nutritional deficiencies, head injury and cirrhosis (reviewed in ref. 7), but cognitive impairment in this population can also occur in their absence.^1^ The mechanisms responsible for this cognitive impairment are not understood. Preventative interventions are limited to thiamine supplementation and relapse prevention. Each medicated detoxification is associated with progressively worsening withdrawal symptoms and progressive cognitive impairment.^8, 9^ Detoxification is thus both a cause for clinical concern, and an important opportunity for intervention.

Microglia are specialized cells of macrophage lineage, highly responsive to their local environment.^10^ They become ‘activated’, a histological term describing the retraction of ramifications and assumption of an amoeboid shape, in response to many stimuli, including tissue damage. In the healthy brain, the microglial activation is modulated by neurons^11^ and basal microglial activation varies regionally. Microglia in the hippocampus express genes that suggest pro-inflammatory priming, even in the absence of disease.^12^

Several functional states of activation are recognized, and two specifically named: an ‘M1’ neurotoxic phenotype that produces pro-inflammatory cytokines, reactive oxygen species^13^ and excitotoxins;^14^ and a neurotrophic ‘M2’ phenotype which secretes anti-inflammatory cytokines, nerve growth factors and clears debris via phagocytosis.^15^ It is hypothesized that further functional types of activated microglia exist^16^ and that the type of activation is contingent both upon the microenvironment,^15^ neuronal regulation^17^ and systemic inflammation.^18^ Either overactivity of the M1 phenotype or suppression of the M2 phenotype could be associated with exacerbation of an acute or chronic neural insult.

Pre-clinical models of alcohol dependence demonstrate microglial activation and expression of inflammatory mediators such as tumor necrosis factor-α (TNFα) and interleukin-6 (IL-6) both in the brain and peripheral blood during alcohol withdrawal.^19, 20^ These changes are associated with neuronal death and learning deficits.^19^ There is evidence that similar processes may occur in humans. An increased density of microglia and increased expression of CCL2, an inflammatory chemokine, are found in the anterior cingulate cortex, midbrain and amygdala of ADPs postmortem.^21^ Pro-inflammatory cytokines including IL-6 and IL-10 are elevated in plasma (reviewed in ref. 22) and increased CCL2 is found in the cerebrospinal fluid^23^ in ADP during withdrawal.

Pre-clinically, hippocampal and piriform cortical microglial activation and proliferation are found during alcohol withdrawal^19, 24, 25^ and can persist for up to 3 weeks.^19, 26^ Microglial activation also is found in the cerebral cortex and cerebellum with chronic alcohol administration in pre-clinical models, and further increased after withdrawal.^24^

Glial cells, including microglia, can be detected *in vivo* in humans using positron emission tomography (PET) radioligands binding to the 18 kDa mitochondrial translocator protein (TSPO), which is highly expressed in activated glia. Although increased TSPO expression is associated with histological cell changes consistent with activation, it does not distinguish between different activation functions, that is M1 or M2 activation. TSPO is also expressed in other central nervous system cell types, including activated astrocytes, endothelium and blood components such as acute phase proteins.^27, 28, 29^ Increased TSPO expression has been demonstrated pre-clinically using [^3^H]PK11195 autoradiography in chronic alcohol intake^24^ and withdrawal models.^19, 26^ [^11^C]PK11195 binding was increased in a small clinical cohort with hepatic encephalopathy,^30^ three members of which had a history of alcohol dependence, though they were long abstinent. Activated microglia may therefore represent a target for intervention around the time of alcohol withdrawal.

The aim of this study was therefore to investigate whether increased TSPO radioligand binding suggesting microglial activation is found in ADP who had recently undergone medically assisted withdrawal. A secondary objective was to explore the relationship between this measure of microglial activation and cognitive function. We hypothesized that increased [^11^C]PBR28 PET signal (measure as a volume of distribution, *V*_T_), would be increased in the brains of ADP particularly in those regions associated with microglial activation in pre-clinical or postmortem samples (cerebellum, hippocampus, midbrain, thalamus and cingulate cortex).^19, 21, 24, 31^ Given that microglial activation has been associated with neuronal damage in pre-clinical models of alcohol withdrawal,^19^ we hypothesized that there would be a negative correlation between TSPO expression and performance on tests of verbal and spatial memory and executive function, which are impaired in alcohol dependence.^1^

## Materials and methods

ADP (DSM-IV) within 1 month of medically assisted withdrawal were recruited through local addiction services, healthy control participants (HC) via local volunteer databases, and HC data obtained from an concurrent study running on the same scanner.^32^ Newly abstinent ADP rather than actively drinking ADP were chosen for inclusion for several reasons. First, the pre-clinical literature predominantly reported increased TSPO expression or microglial activation during and after alcohol withdrawal. Second, scanning actively drinking ADP of the severity encountered in local clinical services is technically challenging, as alcohol intoxication affects PET tracer delivery and the patients are likely to enter withdrawal during scanning, causing tremor, and potentially vomiting and seizures. We scanned people following completion of withdrawal when they were no longer tremulous, no longer taking benzodiazepines and able to tolerate study procedures.

Most ADP had undergone medically assisted detoxification with chlordiazepoxide (*n*=7) or diazepam (*n*=1) before the study. Although none were still taking benzodiazepines at the time of scanning (mean duration since last dose 14 days (range 6–29 days)), three had a positive urine screen for benzodiazepines, reflecting the long metabolite half-life. As metabolites of chlordiazepoxide and diazepam do not bind the TSPO, this was not a concern.^33^ All were prescribed thiamine and vitamin B complex tablets, three acamprosate and one disulfiram.

Exclusion criteria applying to both groups included major physical or psychiatric illness as assessed by the Mini-International Psychiatric Interview (MINI-6),^34^ apart from a history of depression or anxiety, alanine transaminase exceeding five times the upper limit of the normal range, abnormal clotting parameters and consumption of steroids or non-steroidal anti-inflammatory medication. All volunteers were tested for rs6971 TSPO genotype, as the non-synonymous polymorphism at this site leading to a single alanine to threonine substitution affects affinity of [^11^C]PBR28 for TSPO.^35^ Heterozygote ‘Mixed Affinity Binders’ (MABs) have an approximately 50% reduction in the binding relative to the ala:ala homozygotes (‘High Affinity Binders’, HABs)^36^ while threo:threo homozygote ‘Low Affinity Binders’ (LABs) have no detectable signal,^37^ so need to be excluded. In this study, both HABs and MABs were scanned. All the participants were required to produce a negative alcohol breath test on the day of the scan and the alcohol-dependent group to score less than 10 on the Clinical Assessment of Withdrawal from Alcohol scale.^38^ Dependence on other drugs, apart from tobacco, was an exclusion criterion for both groups, but recreational use was allowed in ADP. All the participants gave informed consent about data sharing and both studies received approval from local NHS Research Ethics Committees and ARSAC.

Total lifetime alcohol consumption was assessed in both the groups by the modified Skinner’s questionnaire.^39^ Physiological dependence in ADP was quantified using the Severity of Alcohol Dependence Questionnaire. All the participants completed the Spielberger Trait Anxiety Score, Spielberger State Anxiety Score, Beck Depression Inventory and the Fatigue Severity Scale.^40, 41, 42^ Those controls enrolled in the study investigating alcohol dependence also completed the Obsessive Compulsive Drinking Scale.^43^ Cognitive tests were completed on the day of scanning from a battery previously used by us^1^ including digit span, Trail Making Task A and B,^44^ the Rey-Osterrieth Figure (ROCF)^45^ and Weschler Memory Scale paragraph version (WMS).^46^

Plasma samples were taken at the start of the PET scan in the ADP group for the analysis of diazepam, chlordiazepoxide and their common metabolite desmethyldiazepam, using high-performance liquid chromatography (threshold >4 μg ml^−1^) as the ADP group were recently prescribed benzodiazepines and some tested positive for benzodiazepines on urine drug screen. No controls tested positive for benzodiazepines. The serum samples were analysed in the University of Glasgow for 25 cytokines and chemokines via Luminex human multiplex. High-sensitivity C-reactive protein (0.3–500 mg dl^−1^) was analysed by clinical biochemistry services at the Hammersmith Hospital, London, UK.

The participants were scanned as described previously.^32^ Briefly, each participant received a 90 min [^11^C]PBR28 PET scan following a bolus injection of [^11^C]PBR28 (HC: mean 330.4 MBq, range: 312.4–347.3 MBq; ADP: mean 328.9 MBq, range: 302.7–346.5 MBq), after which dynamic three-dimensional PET data were acquired over 90 min. Continuous arterial blood samples were collected every second from the radial artery for the first 15 min. Discrete blood samples were manually withdrawn at 5, 10, 15, 20, 25, 30, 40, 50, 60, 70, 80 and 90 min after scan start to facilitate measurement of whole-blood and plasma activity. The participants also received a high-resolution T1-weighted magnetic resonance imaging (MRI) scan in a Siemens Verio 3T scanner (Siemens Healthcare, Erlangen, Germany). A consultant neuroradiologist reviewed each MRI scan. Any subjects with clinically significant structural lesions were excluded from the analysis.

The PET images were reconstructed via filtered back projection with attenuation and scatter correction. Dynamic images were separated into 26 frames (8 × 15 s, 3 × 1 min, 5 × 2 min, 5 × 5 min, 5 × 10 min). A metabolite-corrected plasma input function was generated using a method described previously.^32^ The total plasma time activity curve was calculated by multiplying the whole-blood curve by plasma-over-blood ratio, and the parent fraction data were fitted to a sigmoid model:


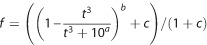


where *t* is time and *a*, *b* and *c* are fitted parameters. The fitted parent fraction profile was multiplied by the total plasma curve and then smoothed post-peak using a tri-exponential fit to derive the required parent plasma input function. A time delay correction was applied to account for delays between blood sample measurement and tomographic tissue measurement.

For analysis, we used the PET data analysis and kinetic modelling toolkit, MIAKAT (www.miakat.org),^47^ which also uses software from SPM5 (Wellcome Trust Centre for Neuroimaging) and FSL (FMRIB, University of Oxford). The PET data were corrected for motion via frame-by-frame co-registration to each participant’s T1 MRI. Anatomical ROIs were delineated by the application of the CIC neuroanatomical atlas,^48^ warped to the participant’s structural MRI scan. The ROIs were applied to the PET data to derive regional time activity curves.

We used a two-tissue compartmental model using a metabolite-corrected input function, with blood volume fixed at 5%, applied to dynamic PET data as described.^49^ Volume of distribution (*V*_T_) was estimated according to the following equation:


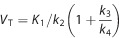


where *V*_T_ is the regional volume of distribution, *K*_1_ and *k*_2_ are rate constants for the movement of [^11^C]PBR28 from plasma to brain parenchyma and parenchyma to plasma respectively, and *k*_3_ and *k*_4_ are rate constants for the movement of [^11^C]PBR28 from the nondisplaceable ligand in the tissue to bound to the specific target, and from bound to nondisplaceable, respectively. As several of the volumes of interest specified *a priori*—hippocampus, midbrain, thalamus—were relatively small volumes adjacent to cerebrospinal fluid, partial volume correction using LoReAN, a hybrid voxel-region-based algorithm,^50^ was undertaken.

Participants’ structural MRI scans were compared using voxel-based morphometry to establish whether there were volumetric differences in ROIs between groups. Following segmentation, segmented tissue maps of each subject were registered to their common average using DARTEL,^51^ then affine registered to the MNI space. The warped images were modulated with the Jacobian determinants of the deformations^52^ and smoothed with an isotropic Gaussian kernel with a full width at half maximum of 8 mm.

The sample size was informed by biomathematical modelling, because of the relative novelty of the tracer and uncertainty about the anticipated diference between groups.^53^ Demographic differences between groups were assessed using unpaired two-tailed *t*-tests applied to parametric data, and Mann–Whitney tests to non-parametric data. Analyses of covariance, implemented in SPSS 20.0 were performed to examine the effect of patient group on [^11^C]PBR28 *V*_T_ across ROIs identified *a priori*. Genotype was included as a fixed factor in the analysis as it is known to influence *V*_T_. Age was included as a covariate as TSPO expression increases with age.^54^ Equality of variance was confirmed using Levene’s test. A two-way analysis of variance was used to investigate whether there were differences in the area under the curve of the input function or in free fraction between groups. A *post hoc* analysis of *V*_T_ in 20 brain regions defined by the CIC atlas (including the frontal lobe, parietal lobe, temporal lobe, occipital lobe, functional subdivisions of these relevant to addiction such as insula and medial prefrontal cortex, thalamus, caudate, putamen, hippocampus, midbrain, pons, medulla and cerebellum) using a mixed-effects model to examine the effects of patient group, region and a patient group × region interaction, corrected for age and genotype, was undertaken. Two-tailed partial correlations, accounting for age and genotype, were performed between hippocampal *V*_T_ and performance on the WMS, ROCF and frontal *V*_T_ and performance on trail making tasks. The primary analysis combined ADP and HC in one group, because of the small sample size. The groups were considered separately in a secondary analysis. A mass univariate general linear model was applied to compare relative grey matter volumes between groups on a voxel-by-voxel basis, accounting for age and intracranial volume, with family-wise error correction (*P*<0.05). Mann–Whitney *U*-tests were used to examine the differences between groups in circulating cytokine concentrations. In a secondary, exploratory analysis, Spearman correlations were performed between individual cytokine concentrations and TSPO binding.

## Results

### Clinical characteristics

The clinical characteristics of the study sample are shown in Table 1. There were no significant group differences in educational attainment between ADP and HC. Most ADP had had at least one previous detoxification (median 1 (range 0–7)) and were moderately to severely dependent (Severity of Alcohol Dependence Questionnaire: 29±9). ADP scored higher on measures of alcohol craving, depression and anxiety, and the Fatigue Severity Scale. ADP performed less well on tests of verbal and spatial memory, but there were no differences in performance on tests related to executive function.

Mean high-sensitivity C-reactive protein was within normal range in both the groups. Albumin was significantly lower in ADP and GGT significantly higher, but other liver function tests were within normal limits in both the groups (see Table 2). No diazepam or chlordiazepoxide was detectable in any blood samples, but desmethyldiazepam was detectable in three ADP (370±148 nm). There was no significant difference (*P*>0.05) in concentrations of any of the cytokines measured (see Table 2).

### Group differences in [^11^C]PBR28 *V*_T_

**Figure 1:**
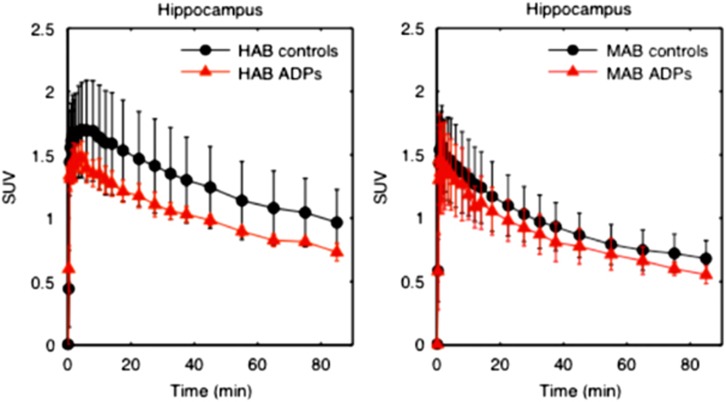
Time activity curves in the hippocampus. This figure shows mean time activity curves with error bars representing standard deviation, from the hippocampus in healthy controls, shown in black, and alcohol-dependent patients (ADPs), shown in red. HAB, high-affinity binder; MAB, mixed-affinity binder; SUV, standardized uptake value.

**Figure 2:**
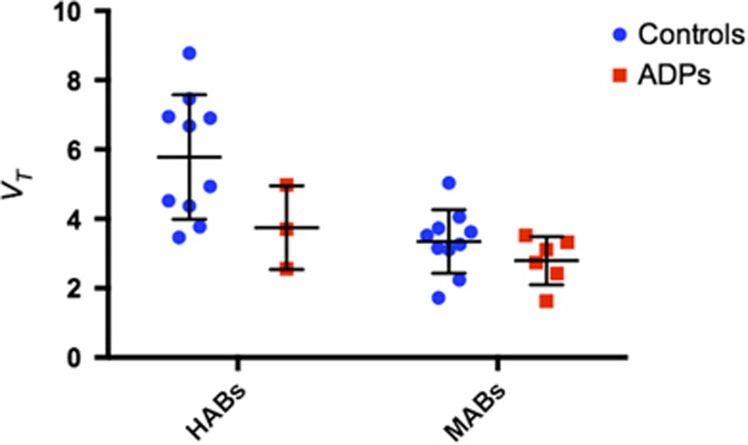
[^11^C]PBR28 *V*_T_ is lower in the hippocampi of alcohol-dependent patients (ADPs) than in controls (mean±s.d.). This figure shows *V*_T_ of individual participants grouped by genotype with ADPs shown as red squares and healthy controls (HCs) as blue circles. The bars show mean and standard deviation in each group. HAB, high-affinity binder; MAB, mixed-affinity binder.

**Figure 3:**
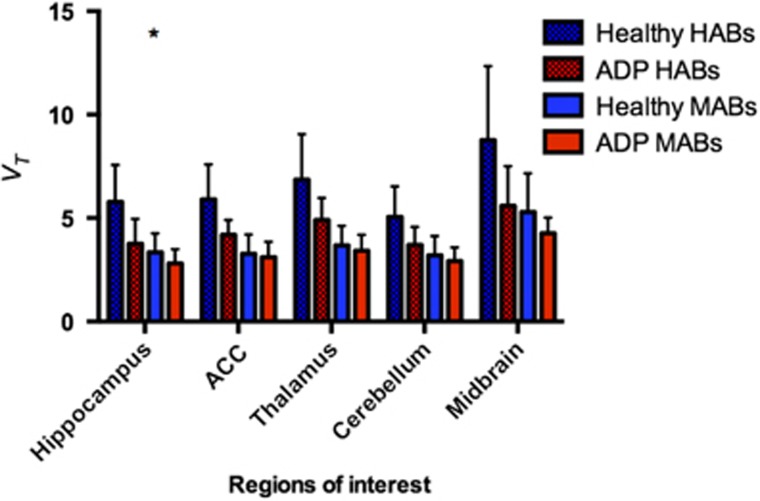
Lower *V*_T_ in regions of interest (ROIs) apart from the hippocampus did not reach significance (mean+s.d.). This figure shows mean *V*_T_ and standard deviation in all the ROIs identified *a priori*. Healthy controls (HCs) are blue and alcohol-dependent patients (ADPs) are red. The bars reflect both patient group and genotype. ACC, anterior cingulate cortex; HAB, high-affinity binder; MAB, mixed-affinity binder.

Hippocampal [^11^C]PBR28 *V*_T_ (corrected for the effects of genotype and age) was 19% lower in ADP relative to HC (F(1,24) 5.694; *P*=0.025; partial *η*^2^=0.192). Group mean time activity curves for the hippocampus are shown in Figure 1, with HABs and MABs shown separately. Mean *V*_T_s in the hippocampus for all genotypes and groups are shown in Figure 2. Differences in other ROIs identified *a priori*—midbrain, thalamus, cerebellum and anterior cingulate cortex—did not reach statistical significance (midbrain: *P*=0.061, partial *η*^2^=0.139; thalamus: *P*=0.091, partial *η*^2^=0.115; cerebellum *P*=0.100, partial *η*^2^=0.108 and anterior cingulate cortex: *P*=0.078, partial *η*^2^=0.124; see Figure 3). The mixed-methods analysis undertaken to interrogate whether there was a general effect of patient group or an interaction between patient group and region, corrected for age and genotype, found no significant effect of patient group (F=1.736; *P*=0.200), but a significant effect of region (F=10.610; *P*<0.001) and a significant region × patient group interaction (F=2.294; *P*=0.002). There were no differences in the area under the curve of the input function between the groups (HAB HC: 2219±334.6; HAB ADP: 2523.8±686.8; MAB HC: 2436±551.9; MAB ADP: 2475±928.5; *P*=0.566) or in free fraction (HAB HC: 0.0247±0.0161; HAB ADP: 0.0115±0.0046; MAB HC: 0.0176±0.0115; MAB ADP: 0.0191±0.0054, *P*=0.424).

### Exploratory correlates of [^11^C]PBR28 *V*_T_

There were no significant correlations between peripheral cytokine concentrations and [^11^C]PBR28 *V*_T_. Age and genotype were significantly associated with variation in [^11^C]PBR28 *V*_T_ in all the brain regions tested (for example, in the hippocampus: genotype F(1,24)=8.190; *P*=0.009; age: F(1,24)=5.370; *P*=0.029). There was no patient group × genotype interaction (*P*>0.05). *V*_T_ was not associated with duration of abstinence in alcohol-dependent group in any ROI. Voxel-based morphometry conducted using the MRI structural images to establish whether there was atrophy in ADP revealed no significant differences between the groups (family-wise error corrected *P*>0.05; t-statistic threshold=6.01). The peak t-statistics for the ROIs identified *a priori* were as follows: anterior cingulate cortex: 1.805; left hippocampus 1.680; right hippocampus: 1.447; midbrain 2.885; left thalamus: 2.031; right thalamus: 2.840; cerebellum: 2.312.

### Hippocampal [^11^C]PBR28 *V*_T_ and cognitive performance measures

**Figure 4:**
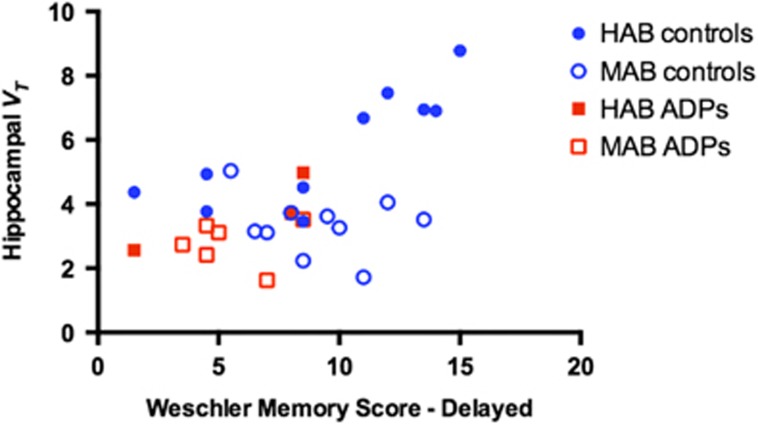
Correlation between hippocampal *V*_T_ and verbal memory. This figure shows the positive relationship between hippocampal binding and performance on a verbal memory task. Healthy controls (HCs) are blue and alcohol-dependent patients (ADPs) are red. Filled squares/dots are HABs and unfilled squares/dots are MABs. HAB, high-affinity binder; MAB, mixed-affinity binder.

There was a positive correlation (after controlling for the effects of age and genotype) between hippocampal *V*_T_ and both WMS and ROCF performance (WMS: *r*=0.720, *P*<0.001; ROCF: *r*=0.541; *P*=0.004; see Figure 4) when ADP and HC were combined in one group. A positive correlation was also found in the HC group alone between hippocampal *V*_T_ and scores on the delayed WMS (*r*=0.738; *P*<0.001),^46^ but not scores on the delayed ROCF test^45^ (*r*=0.445; *P*=0.073). The ADP clustered at the lower end of the distribution of HC in the combined analysis but the correlation with verbal memory was not significant in the ADP group alone (*r*=0.331; *P*=0.468). There were no associations between hippocampal *V*_T_, and performance on digit span or the trail making task, nor any associations between the frontal cortex *V*_T_ and performance on digit span or the trail making task.

## Discussion

To the best of our knowledge, this is the first study to report changes in [^11^C]PBR28 *V*_T_ in a cohort of otherwise healthy ADPs within the first few weeks of abstinence. Refuting our initial hypothesis, we found that [^11^C]PBR28 *V*_T_ is decreased in the hippocampus in recently abstinent ADPs relative to healthy participants with no significant changes in other brain ROIs identified *a priori*. In an exploratory analysis, we also found a positive relationship between hippocampal [^11^C]PBR28 *V*_T_ and verbal memory in the healthy participants. When the patient and healthy control group were combined, the correlation remained and a significant positive correlation was found between hippocampal [^11^C]PBR28 *V*_T_ and both verbal and spatial memory.

We hypothesized that [^11^C]PBR28 binding would be increased in alcohol withdrawal, based on pre-clinical evidence.^19, 20, 25, 26^ However, our findings of a decrease are more consistent with either a decrease in expression of the protein or a loss of cells expressing the protein. TSPO is a mitochondrial protein and would therefore be expected to decrease with a reduction of mitochondrial density. Slight decreases in mitochondrial enzymes and uncoupling with production of potentially damaging free radicals have been reported in a pre-clinical chronic alcohol model.^55^

Alternatively, decreases in the activation state, or absolute numbers of several populations of cells could explain the reduction seen. First, loss, or altered activation, of microglia or astrocytes could explain the change. The one existing stereological human postmortem study of microglia and astrocytes in ADP, which included data on the hippocampus supports this as there was selective loss of both astrocytes and microglia.^56^ Prior human magnetic resonance spectroscopic results showed no decreases in myo-inositol, a metabolic marker for activated astrocytes, in recently abstinent alcoholics^57, 58^ making astrocytic changes around the time of withdrawal less likely than microglial changes. Pre-clinically, microglial activation during alcohol withdrawal has been reported to be M2 activation, raising the possibility that loss of such activation may hamper repair.^26^ Second, lower [^11^C]PBR28 binding could relate to suppressed neurogenesis, as TSPO is also expressed by hippocampal neural stem cells.^59^ Pre-clinical findings regarding the effect of alcohol on neurogenesis have yet to be replicated in human postmortem samples, which showed no differences in numbers of neural progenitors between ADP and HC in the subventricular zone and olfactory bulb.^60^ Absolute numbers of neural progenitors are low in adult humans, making this unlikely.^60^ Finally, TSPO is also expressed in the endothelial cells,^61^ so microvascular changes could contribute to our findings. Changes in the hippocampal capillary lumen diameter and decrease in the length and density has been described in at least one pre-clinical chronic alcohol model.^62^

The difference between pre-clinical findings and the outcome of this clinical study may relate to differences between pre-clinical models of alcohol dependence and clinical populations. Pre-clinical studies are performed in adolescent rodents, and periods of heavy alcohol exposure are relatively short (<2 weeks). For logistical reasons, we were not able to scan patients during the first week of withdrawal. Some pre-clinical models show only very short-lived microglial activation,^26, 63^ but others show activation at 3 weeks of abstinence.^19^ There was no relationship between duration of abstinence and [^11^C]PBR28 *V*_T_ in our study, though it was not designed to investigate this. Of interest is that while pre-clinical studies in cocaine dependence have reported pro-inflammatory gene expression,^64^ clinical imaging has shown no difference in TSPO expression,^65^ suggesting there may be similar translational challenges across addictions.

Clinical populations in the United Kingdom are medicated with benzodiazepines during withdrawal. Suppression of microglial activation via GABA(A) transmission has been reported raising the possibility that microglial suppression by benzodiazepines explains the decrease.^66^ A direct effect of benzodiazepines binding the TSPO is however unlikely as only diazepam at high dose binds the TSPO and the only benzodiazepine present in the ADP plasma was the metabolite desmethyldiazepam, which does not bind the TSPO.^33^

We found that hippocampal [^11^C]PBR28 binding was positively associated with verbal memory in the sample considered as a whole and in healthy controls. Although ADPs clustered towards the lower end of the distribution, the relationship was not maintained in ADPs alone—probably because of small numbers, particularly small numbers of HABs, who have a higher signal-to-noise ratio. To detect a correlation of the strength of that seen in HC with 80% power, 11 HABs would have been needed.

The positive correlation between TSPO expression and memory could be attributed to the function of the protein itself or the cells which express it. TSPO over-expression has been found pre-clinically to protect against lipopolysaccharide induced memory dysfunction.^67^ The relationship between mitochondrial function, TSPO expression and memory function under healthy conditions has not been explored. Microglia and astrocytes are both involved in memory function in the healthy hippocampus: while microglia integrate neural progenitors into hippocampal circuits and undertake activity-related synaptic remodelling (reviewed in ref. 68), astrocytic release glutamate, adenosine triphosphate and cytokines helps to consolidate nascent synaptic connections.^69^ The positive correlation seen in healthy participants supports the importance of microglia and astrocytes, and perhaps TSPO itself, in the healthy functioning of the hippocampus.

Although we did not find a correlation in the ADPs alone, the finding of a positive relationship between memory performance and TSPO expression is intriguing. Verbal and spatial memory deficits, which relate to hippocampal function, are well described in alcohol dependence.^1, 70, 71^ We did not find brain volume changes in our small ADP sample, but larger volumetric studies have shown hippocampal atrophy in alcohol-dependent cohorts.^72, 73, 74, 75^ No relationship between volume loss and memory has been demonstrated, raising the possibility that loss or dysfunction of cell subsets such as microglia or astrocytes is a more important determinant.

The moderately to severely dependent drinkers that we studied were challenging to recruit and retain as they were relatively unstable and at high risk of disengagement from treatment. Recruitment challenges affected both statistical power to detect the group differences and stringency of inclusion and exclusion criteria. The study is therefore affected by limitations mainly around size and design. Our patient sample is small, was scanned on average 3 weeks from cessation of alcohol, were receiving relapse prevention medications and were more likely to be smokers. The small sample meant we were unable to interrogate the possible contribution of these factors. Another limitation is that the majority of the patient group had undergone medicated detoxification, raising the possibility that changes are seen related to medications taken during detoxification or other non-specific effects.

We found a statistically significant decrease in [^11^C]PBR28 *V*_T_ in the hippocampus of ADPs shortly following alcohol withdrawal consistent with microglial or astrocytic loss or functional change, or changes in TSPO expression related to oxidative stress or mitochondrial pathology. This finding is supported by a previous postmortem study showing hippocampal microglial and astrocytic loss in ADP. Hippocampal [^11^C]PBR28 *V*_T_ indicative of TSPO expression was positively correlated with performance on a delayed memory task, suggesting this may be of clinical relevance. This relates to pre-clinical research suggesting that microglial activation may be related to homeostatic functions rather than inflammatory functions in the hippocampus under healthy conditions. Binding was not higher in the alcohol-dependent participants, raising questions about microglial and mitochondrial function in this context and how this may translate into treatment targets. It remains to be established whether an increase in TSPO expression occurs in humans during chronic drinking, or in binge alcohol exposure, which more closely resembles animal models.

## Figures and Tables

**Table 1:** Characteristics of the sample

	*Alcohol-dependent*	*Control*	P
*Demographic data*			
* N*	9	20	
Age	45±13	45±7	0.590
Male:female	9:0	14:5	
TSPO genotype (HABs:MABs)	3:6	10:10	
Lifetime dose (kg)	83.29 (14.57–273.96)	10.91 (0–39.92)	0.005**
Alcohol use over past 28 days/28 days before detoxification (g)	5616 (1680–10 080)	244 (0–1080)	<0.001**
Current cigarette smoking (*n*)	8	5	
Illicit drug use	4	0	
Past history of depression	4	2	
Severity of Alcohol Dependence Questionnaire	29±9		
Obsessive Compulsive Drinking Scale	12.4±8.0	3.9±2.4	0.003**
Beck Depression Inventory	11±6	4±4	0.002**
Spielberger Trait Anxiety Inventory	44±6	35±11	0.015*
Spielberger State Anxiety Inventory	33±10	29±9	0.013*
Fatigue Severity Scale	4±0.7	2±1.2	0.004**
			
*Cognitive tests*			
Weschler Memory Scale Immediate	8±2.9	11±2.8	0.027*
Weschler Memory Scale Delayed	6±2.5	9±3.6	0.013*
Rey-Osterrieth Complex Figure Immediate	14±11.2	20±8.4	0.147
Rey-Osterrieth Complex Figure Delayed	14±9	21±7.9	0.048*
Trails A (time to complete)	30.3±9.30	29.32±16.9	0.861
Trails B (time to complete)	62.8±36.48	57.7±30.25	0.700
Digit span	16.0±3.12	17.6±4.52	0.340

Abbreviations: HAB, High Affinity Binders; MAB, Mixed Affinity Binders; TSPO, translocator protein. Parametrically distributed data are presented as mean±s.d. Non-parametrically distributed data are presented as median (range). *P<0.05; **P<0.01. Illicit drug use in alcohol group: two participants used cocaine and two used cannabis.

**Table 2:** Blood results in ADP and HC

	*Alcohol-dependent*	*Control*	P
*Standard clinical blood tests*			
Haemoglobin	14.8±1.2	14.6±1.2	0.731
Mean cell volume	96.1±6.1	84.9±13.6	0.007**
White cell count	9.3±2.0	6.5±1.3	0.002**
Bilirubin	8±5	12±8	0.161
Alanine transaminase	56±20	26±8	0.110
Alkaline phosphatase	71±50	68±19	0.702
Gamma glutamyl transferase	134 (range 15–466)	30±20	0.064
Albumin	40±3	44±3	0.004**
Adjusted partial thromboplastin time	26.6±2.3	28±2	0.086
C-reactive protein	3.0±2.39	2.6±3.63	0.775
			
*Pro- and anti-inflammatory cytokines*			
Tumour-necrosis factor α	0 (0–225.29)	0 (0–859.03)	0.776
Interleukin 1β	0 (0–414.77)	0 (0–1288.16)	0.776
Interleukin 1 receptor antibody	0 (0–518.83)	0 (0–1696.38)	0.776
Interleukin 6	0 (0–285.06)	0 (0–902.75)	0.882
Interleukin 10	130.31 (60.68–537.95)	74.00 (42.50–1283.45)	0.412
GM-CSF	61.64 (26.99–387.18)	41.69 (11.77–1173.56)	0.370
			
*Type 1 interferons*			
Interferon α	0 (0–305.73)	0 (0–902.75)	0.824
			
*Cytokines related to T-cell activation*			
IL-2	0 (0–292.86)	0 (0–866.97)	0.710
IL-7	32.89 (23.84–64.53)	36.60 (17.88–359.04)	0.552
IL-15	0 (0–916.53)	0 (0–2933.43)	0.766
IL-2R	21.87 (2.28–227.93)	12.15 (4.84–595.21)	1.000
			
*Th1 cytokines*			
IL-12	10 019 (4770–46 685)	10 205 (8258–29 077)	0.412
IFN-γ	0 (0–225.29)	0 (0–859.03)	0.766
			
*Th2 cytokines*			
IL-5	5.06 (3.51–560.75)	5.93 (3.1–1667.44)	1.000
IL-13	39.53 (0–1357.48)	26.59 (0–4083.75)	0.766
			
*Th17 cytokines*			
IL-17	22.89 (15.64–663.44)	19.39 (15.64–2174.24)	0.552
			
*Chemokines*			
CXCL-8 (IL-8)	20.16 (10.68–1439.79)	11.6 (2.91–2808.70)	0.131
CXCL-9 (MIG)	0 (0–1698.11)	0 (0–5066.90)	0.766
CXCL-10 (IP-10)	170.46 (60.82–1088.97)	82.56 (0–3133.54)	0.131
CCL-2 (MCP-1)	0 (0–196.47)	0 (0–663.28)	0.710
CCL-3 (MIP-1α)	0.29 (0.19–595.03)	0.29 (0.19–2204.86)	0.824
CCL-4 (MIP-1β)	855.78 (218.01–2023.38)	399.59 (160.48–3672.07)	0.201
CCL-5 (RANTES)	189.59 (48.06–637.74)	105.53 (45.23–609.39)	0.295
CCL-11 (Eotaxin)	19.39 (10.56–839.90)	22.11 (7.41–2334.10)	0.656

Abbreviations: ADP, alcohol-dependent patients; GM-CSF, gray matter-cerebrospinal fluid; HC, healthy controls; IFN, interferon; IL, interleukin. For parametrically distributed data, mean±s.d. is displayed. For non-parametrically distributed data, median (range) is displayed. **P<0.01.
